# Takotsubo cardiomyopathy in myasthenia gravis: a systematic review of case reports with subtype-based analysis

**DOI:** 10.1186/s12245-026-01145-w

**Published:** 2026-02-12

**Authors:** Hideya Itagaki, Takuro Hagino, Tomoyuki Endo

**Affiliations:** 1 Division of Emergency and Disaster Medicine, Tohoku Medical and Pharmaceutical University Hospital, 1-12-1 Fukumuro, Miyagino-ku, Sendai, Miyagi 983-8512 Japan; 2 Division of Neurology, Tohoku Medical and Pharmaceutical University Hospital, 1-12-1 Fukumuro, Miyagino-ku, Sendai, Miyagi 983-8512 Japan

**Keywords:** Myasthenia gravis, Takotsubo cardiomyopathy, Myasthenic crisis, Stress cardiomyopathy, Apical ballooning, Review

## Abstract

**Background:**

Takotsubo cardiomyopathy (TTC) is a stress-induced cardiac disorder characterized by transient left ventricular dysfunction. Myasthenia gravis (MG), an autoimmune neuromuscular disease, can precipitate TTC during exacerbations such as myasthenic crises. However, the clinical features and outcomes of TTC associated with MG remain unclear.

**Methods:**

We conducted a systematic review by the PRISMA guidelines. A comprehensive search of PubMed, Web of Science, and Google Scholar was performed up to May 31, 2025, using terms related to TTC and MG. Case reports and series were included if they described patients with confirmed diagnoses of both MG and TTC, with MG exacerbation occurring at the time of TTC onset. Data were extracted and compared between apical and non-apical TTC subtypes. Statistical analyses included the Mann-Whitney U test and chi-squared or Fisher’s exact test, as appropriate.

**Results:**

A total of 38 articles comprising 40 cases were included. The median age was 69 years; 67.5% of the participants were female. Apical TTC was the most common subtype (71.8%). Myasthenic crisis was the most frequent TTC trigger (37.5%), while chest pain was reported in only 17.5% of cases. Mechanical ventilation and ICU admission were required in 95% and 97.4% of cases, respectively. Overall mortality was 17.5%. No significant differences were found between TTC subtypes; apical cases showed numerically higher troponin levels, which should be interpreted cautiously.

**Conclusions:**

TTC associated with MG exacerbation is a rare but serious condition with high rates of respiratory failure, ICU admission, and mortality. Emergency and critical care physicians should suspect TTC in MG patients presenting with hemodynamic instability or ECG abnormalities, even in the absence of chest pain.

**Supplementary Information:**

The online version contains supplementary material available at 10.1186/s12245-026-01145-w.

## Background

Myasthenia gravis (MG) is an autoimmune disorder in which autoantibodies are produced against molecules located on the postsynaptic membrane of the neuromuscular junction, such as acetylcholine receptors (AChR) and muscle-specific receptor tyrosine kinase (MuSK), thereby impairing signal transmission from nerves to muscles [1]. According to some reports, approximately 16% of patients with MG have concomitant cardiac involvement, including arrhythmia, pericarditis, and myocarditis [2–7]. Among these, rare cases have been reported in which MG triggers the onset of Takotsubo cardiomyopathy (TTC) [3].

TTC is characterized by transient systolic and diastolic left ventricular dysfunction with regional wall motion abnormalities. It predominantly affects older women and is often preceded by emotional or physical stressors [8]. Although the pathophysiology of TTC has not been fully elucidated, it is believed that acute emotional or physical stress leads to excessive activation of the sympathetic nervous system. This results in the release of norepinephrine from sympathetic nerve terminals in the heart, as well as the secretion of both epinephrine and norepinephrine from the adrenal medulla [1]. These catecholamines are thought to induce coronary and peripheral vasospasm, leading to transient myocardial injury and resulting in left ventricular wall motion abnormalities [2–4].

Similarly, in MG, stressors such as infection, trauma, emotional distress, or certain medications are known to trigger a catecholamine surge, which can precipitate a myasthenic crisis [5, 6]. While the exact mechanism of TTC onset during a myasthenic crisis remains unclear, the triggers for both conditions often overlap. It is therefore hypothesized that TTC and myasthenic crisis may occur simultaneously or sequentially through shared stress-related pathophysiological mechanisms [7, 8].

Given this background, TTC occurring in the setting of MG represents a clinically important but underrecognized condition that requires early recognition and appropriate management in emergency and intensive care settings. Since the publication of the previous systematic review in 2019, a substantial number of additional cases of TTC associated with MG have been reported, allowing for a more comprehensive and updated analysis.

Therefore, the aims of this systematic review were to [1] update the existing literature by including recently published cases up to May 2025 [2], comprehensively describe the clinical characteristics, management, and outcomes of TTC associated with MG, and [3] compare apical and non-apical TTC subtypes to explore potential differences in presentation, critical care requirements, and prognosis.

## Methods

### Protocol and registration

This systematic review was conducted in accordance with the PRISMA (Preferred Reporting Items for Systematic Reviews and Meta-Analyses) checklist. The review protocol was not pre-registered on PROSPERO or any other platform.

### Eligibility criteria

We included studies published up to May 31, 2025 that met all the following criteria: [1] diagnosis of MG confirmed by serological or electrophysiological testing; [2] diagnosis of TTC confirmed by echocardiography and/or coronary angiography, with transient regional wall motion abnormalities consistent with TTC; and [3] confirmed exacerbation of MG (including myasthenic crisis) at the time of TTC onset. Studies that were not case reports or case series, or that had not undergone peer review, were excluded. No language restrictions were applied. Non-English articles were included, and translations were performed using both automated tools and expert translators to ensure accuracy.

### Information sources and search strategy

We conducted a comprehensive literature search using PubMed, Web of Science, and Google Scholar databases up to May 31, 2025. For PubMed, the following search string was used: ((“Takotsubo cardiomyopathy” OR “Takotsubo syndrome” OR “Broken heart syndrome” OR “Stress-induced cardiomyopathy” OR “Stress cardiomyopathy”) AND (“Myasthenia gravis” OR “Myasthenic crisis”)). For Web of Science and Google Scholar, equivalent search strategies were adapted using the same combinations of keywords related to Takotsubo cardiomyopathy and myasthenia gravis, including “Takotsubo cardiomyopathy,” “Takotsubo syndrome,” “broken heart syndrome,” “stress-induced cardiomyopathy,” and “stress cardiomyopathy,” combined with “myasthenia gravis” or “myasthenic crisis.” In Google Scholar, additional filters were applied to prioritize case reports and case series by including the terms “case report” or “case series” and excluding conference proceedings and books where applicable. Given the limited reproducibility of Google Scholar searches, multiple keyword combinations were used to maximize sensitivity. The search yielded 459 records from PubMed, 235 from Web of Science, and 1,547 from Google Scholar. Furthermore, manual screening of relevant articles was conducted to identify additional eligible studies.

### Study selection

Two reviewers (HI and TH) independently conducted the initial screening of titles and abstracts. Full-text articles were then reviewed by the same two authors, and those not meeting the inclusion/exclusion criteria were excluded. Any disagreements regarding study eligibility were resolved through discussion and consensus between the two reviewers.

### Data collection process and data item

The following data were extracted from each included study: patient demographics (age, sex), comorbidities, MG clinical features (antibody status, thymoma, and treatments), TTC characteristics (subtype, ejection fraction, ECG findings, and troponin levels), and critical care outcomes (mechanical ventilation, ICU admission, and mortality).

### Quality assessment

The quality of the included case reports was evaluated using the CARE (CAse REport) guidelines [9]. Each item on the 30-point CARE checklist was scored as 1 if adequately reported, 0 if not mentioned, or 0 if unclear. Two reviewers independently assessed the CARE score for each report, and any discrepancies were resolved through discussion to achieve consensus, ensuring objectivity and reproducibility.

### Definitions and diagnostic criteria

Takotsubo cardiomyopathy was diagnosed based on the criteria in each original report, primarily using echocardiography or left ventriculography to confirm transient regional wall motion abnormalities (RWMA). Most cases met established diagnostic frameworks, such as InterTAK criteria, characterized by RWMA extending beyond a single coronary territory in the absence of alternative causes [10].

TTC subtypes (apical, midventricular, basal, and mixed) were classified based on imaging patterns, primarily using transthoracic echocardiography with left ventriculography as a complementary modality [11]. To exclude obstructive coronary artery disease, coronary angiography (CAG) or left heart catheterization was performed in 65% of cases (26/40). When CAG was not performed, ACS was excluded based on clinical assessment, electrocardiographic findings, cardiac biomarker profiles, and noninvasive imaging [12]. These data are summarized in Supplementary Table S1.

While cardiac magnetic resonance imaging (MRI) is ideal for differentiating Takotsubo cardiomyopathy from myocarditis through late gadolinium enhancement (LGE) patterns, it was rarely performed in the reviewed cases [13]. Consequently, myocarditis was excluded based on clinical presentation and biomarkers rather than advanced imaging or histopathology.

### Synthesis of results

The extracted cases were organized into a tabular format, summarizing clinical characteristics and outcomes. A narrative synthesis was performed, and clinical characteristics were compared between the Apical and Non-Apical types of TTC to explore potential differences.

### Data analysis

All statistical analyses were performed for exploratory and descriptive purposes only. For continuous variables that were not normally distributed, the Mann–Whitney U test was used; for categorical variables, the Pearson chi-squared test or Fisher’s exact test was employed. Given that this review is based exclusively on case reports and case series, the study was not powered to detect statistically meaningful differences between subgroups. Therefore, inferential statistical testing should be interpreted with caution, and the analyses should be regarded as hypothesis-generating rather than confirmatory. Descriptive statistics were reported as medians with interquartile ranges (IQR) for continuous variables and as frequencies and percentages for categorical variables. All analyses were conducted using Stata/MP 18.0 (Stata Corp, College Station, Texas, USA).

## Results

**Fig. 1:**
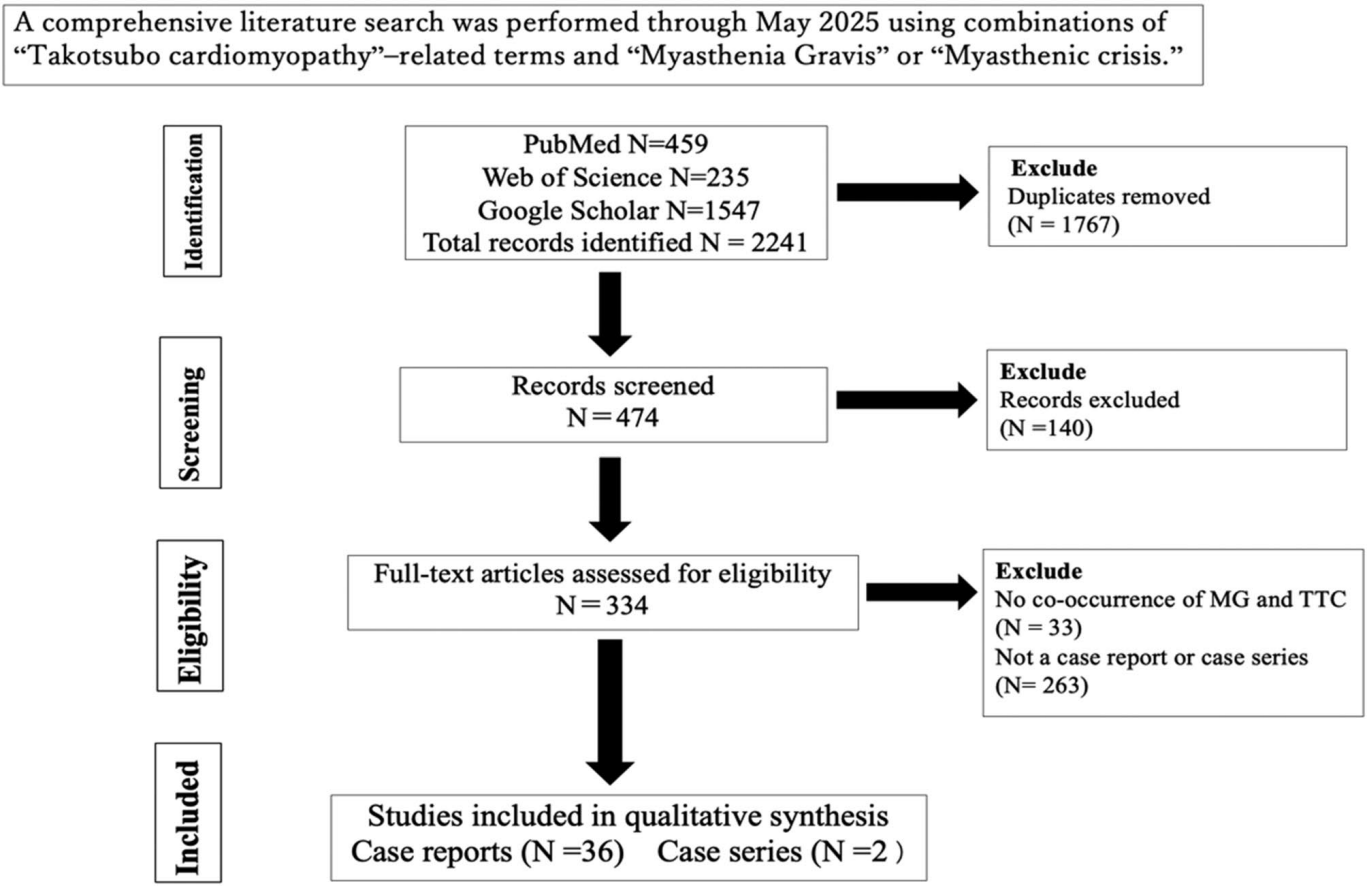
PRISMA flow diagram of study selection. Legend: Flowchart illustrating the process of study identification, screening, eligibility, and inclusion for the systematic review

All subgroup analyses were exploratory and should be interpreted cautiously given the limited sample size. A total of 2,241 articles were retrieved from PubMed, Web of Science, and Google Scholar (Fig. 1).

After removal of duplicates, 474 records remained and were screened by title and abstract. Of these, 140 records were excluded. The full texts of the remaining 334 articles were assessed for eligibility. Among them, 296 were excluded (33 did not report co-occurrence of MG and TTC; 263 were not case reports or case series). Ultimately, 38 studies (36 case reports and 2 case series) were included in this review. The average CARE score for the selected reports was 20, indicating moderate overall reporting quality. The highest score was 25, and the lowest was 11 out of a maximum of 30 points.

### Patient characteristics

This review included 40 patients with coexisting MG and TTC reported across 38 articles [8, 14–50]. Table 1 summarizes the patient demographics.

**Table 1:** Clinical characteristics of patients with MG and TTC

Item	Published cases	NA
	*N* = 40	
**Age**	69(58 ~ 76.5)	0
**Sex, No. (%)**		0
Male	13(32.5%)	
Female	27(67.5%)	
**Past medical history, No. (%)**	25(62.5%)	0
Hypertension	12(30%)	
Diabetes Mellitus	7(17.5%)	
Thyroid disease	6(15%)	
COPD	4(10%)	
**Thymoma, No. (%)**		1
Present	6(15.4%)	
Absent	30(76.9%)	
Post-Thymectomy	3(7.7%)	
**Chief complaint, No. (%)**		0
Dyspnea	33(82.5%)	
Dysphagia	14(35%)	
Muscle weakness	7(17.5%)	
Chest pain	7(17.5%)	
Dysarthria	6(15%)	
Ptosis	4(10%)	
Diplopia	2(5%)	

Summary of age, sex, comorbidities, thymoma status, and presenting symptoms in 40 reported cases of Takotsubo cardiomyopathy associated with myasthenia gravis

The median age was 69 years (IQR: 58–76.5), and 28 patients (67.5%) were female. Past medical history included hypertension in 12 cases (30%), diabetes mellitus in 7 (17.5%), thyroid disease in 6 (15%), and COPD in 4 (10%). Sixteen patients (40%) had no notable comorbidities. Thymoma was present in 9 cases (23%), including those with a history of thymectomy. The most common presenting symptoms were dyspnea (82.5%), dysphagia (35%), muscle weakness (17.5%), chest pain (17.5%), dysarthria (15%), ptosis (10%), and diplopia (5%).

### Characteristics of Myasthenia Gravis

Table 2 outlines the clinical features of MG.

**Table 2:** Clinical features and treatment of myasthenia Gravis prior to TTC onset

Item	Published cases	NA
	*N* = 40	
**Type of MG, No. (%)**		3
Generalized MG	35(94.6%)	
Ocular MG	2(5.4%)	
**Antibody Type, No. (%)**		13
Anti-AChR antibody	24(88.9%)	
Striational antibody	4(11.1%)	
**Pre-TTC MG Treatment, No. (%)**		3
Cholinesterase inhibitors	21(57.8%)	
Corticosteroids	14(37.8%)	
Immunosuppressive agents	4(10.8%)	
Immunoglobulin	4(10.8%)	

MG subtype, antibody status, and medications used prior to TTC onset, including cholinesterase inhibitors, corticosteroids, immunosuppressants, and intravenous immunoglobulin

Generalized MG was observed in 35 patients (94.6%), and ocular MG in 2. Anti-AChR antibodies were positive in 24 cases (88.9%), while MuSK antibodies were not detected. Striational antibodies were reported in 4 cases. Regarding pre-TTC MG treatment, cholinesterase inhibitors were used in 21 cases (57.8%), corticosteroids in 14 cases (37.8%), intravenous immunoglobulin (IVIG) in 4 cases (10.8%), and immunosuppressive agents, such as tacrolimus or azathioprine, in 4 cases (10.8%). Many cases were managed primarily with cholinesterase inhibitors.

### Characteristics of Takotsubo cardiomyopathy

Table 3 shows the characteristics of TTC.

**Table 3:** Characteristics of Takotsubo cardiomyopathy in patients with MG

Item	Published cases	NA
	*N* = 40	
TTC Type, No. (%)		1
Apical type	28(71.8%)	
Mixed type	5(12.8%)	
Midventricular type	4(10.3%)	
Basal type	2(5%)	
**EF, No. (%)**		10
30%< EF	16(53.3%)	
EF ≦ 30%	14(46.7%)	
**Electrocardiographic abnormality, No. (%)**		
ST Elevation	20(50%)	
ST Depression	4(10%)	
T-wave Inversion	12(30%)	
QT Prolongation	6(15%)	
Abnormal Q Wave	2(5%)	
VT	1(2.5%)	
Af	4(10%)	
**Troponin elevation**	34(97.1%)	5
**Troponin (IQR) [ng/mL]**	1.83(0.67 ~ 3.20)	5

Distribution of TTC subtypes, left ventricular ejection fraction, ECG findings, and troponin levels among patients with MG-associated TTC

The most frequent subtype was the typical apical type (28 cases, 71.8%), followed by the mixed type (5 cases), the midventricular type (4 cases), and the basal type (2 cases). Left ventricular ejection fraction (EF) was reduced in many patients, with 14 cases (46.7%) showing EF ≤ 30%. Electrocardiographic abnormalities were common: ST-segment elevation in 20 cases (51.3%), ST depression in 4 cases, T wave inversion in 12 cases, QT prolongation in 6 cases, and arrhythmias (e.g., atrial fibrillation or ventricular tachycardia) in 5 cases. Troponin elevation was observed in over 90% of the patients tested, with a median value of 1.83 ng/mL (interquartile range, 0.67–3.20 ng/mL).

### Clinical course, treatment, and outcomes

Table 4 shows the incidence of MG crisis, treatment details, ICU management, and outcomes in cases of MG-TTC co-occurrence.

**Table 4:** Clinical course, treatment, and outcomes of MG-TTC co-occurrence

Item	Published cases	NA
	*N* = 40	
**Presence of Myasthenic Crisis, No. (%)**	38(95%)	0
**Treatment for myasthenic exacerbation, No. (%)**		1
Steroid pulse therapy	4(10.3%)	
Plasma exchange	14(35.9%)	
Adjustment of cholinesterase inhibitors	23(59%)	
Initiation or addition of immunosuppressive agents	9(23.1%)	
Initiation or escalation of corticosteroids	27(69.2%)	
Intravenous immunoglobulin	21(53.8%)	
Thymectomy	2(5.1%)	
**Circulatory support, No. (%)**		7
Vasopressors	14(42.4%)	
norepinephrine	6(18.2%)	
dobutamine	4(12.1%)	
dopamine	3(9.1%)	
Pacemaker	2(6.1%)	
Intra-aortic balloon pumping (IABP)	1(3%)	
No support	12(36.4%)	
**Mechanical ventilation, No. (%)**	38(95%)	0
**ICU admission, No. (%)**	38(97.4%)	1
**Outcome, No. (%)**		
Recovery	33(82.5%)	0
Death	7(17.5%)	

Frequency of MG crisis, treatment, circulatory and ventilatory support, ICU admission, and patient outcomes in the analyzed cases

Among the 40 reported cases, the incidence of MG crisis was 95%, with most cases experiencing a crisis.MG exacerbations were treated with corticosteroid initiation or escalation in 69.2%, adjustment of cholinesterase inhibitors in 59%, IVIG in 53.8%, plasma exchange in 35.9%, and initiation or addition of immunosuppressive agents in 23.1%. Steroid pulse therapy was used in 10.3% of cases, and thymectomy in 5.1%. Circulatory support was required in 42.4% of cases: norepinephrine in 18.2%, dobutamine in 12.1%, and dopamine in 9.1%. Pacemaker support was provided in 6.1%, and intra-aortic balloon pumping (IABP) in 3%. In contrast, 36.4% of patients did not require circulatory support. Mechanical ventilation was required in 38 patients (95%), and ICU admission occurred in 38 patients (97.4%). Outcomes showed that 33 patients (82.5%) survived and were discharged, while 7 (17.5%) died.

### Comparison by TTC type

We classified TTC cases into apical type (*n* = 28) and non-apical type (*n* = 11), and compared the clinical characteristics between the groups (Table 5).

**Table 5:** Comparison between Apical and Non-Apical TTC subtypes

Item	Apical(*N* = 28)	Non-Apical(*N* = 11)	*p*
**Age**	69.5(IQR 21)	66(IQR 16)	0.492
**Sex, No. (%)**			1
Male	9(32.1%)	3(27.3%)	
Female	19(67.9%)	8(72.7%)	
**Pre-TTC MG Treatment, No. (%)**			
Cholinesterase inhibitors	13(50%)	7(70%)	0.456
Corticosteroids	8(30.8%)	6(60%)	0.14
Immunosuppressive agents	2(7.7%)	1(10%)	1
Immunoglobulin	2(7.7%)	2(20%)	0.305
**EF, No. (%)**			0.7
30%< EF	11(55%)	4(44.4%)	
EF ≦ 30%	9(45%)	5(55.6%)	
**Troponin [ng/mL]**	2.47(IQR 2.927)	0.814(IQR 1.55)	0.06
**TTC trigger, No. (%)**			0.432
MG crisis	11(39.3%)	4(36.4%)	
Psychological stress	2(7.1%)	0	
Physical stress	11(39.3%)	3(27.3%)	
Iatrogenic	3(10.7%)	4(36.4%)	
No known trigger	1(3.6%)	0	
**Treatment for myasthenic exacerbation, No. (%)**			
Steroid pulse therapy	3(11.1%)	1(9.1%)	1
Plasma exchange	11(40.7%)	3(27.3%)	0.488
Adjustment of cholinesterase inhibitors	18(66.7%)	5(45.5%)	0.285
Initiation or addition of immunosuppressive agents	7(25.9%)	1(9.1%)	0.395
Initiation or escalation of corticosteroids	20(74.1%)	5(45.5%)	0.135
Intravenous immunoglobulin	14(51.9%)	6(54.6%)	1
**Circulatory support, No. (%)**			
Vasopressors	10(43.5%)	4(50%)	1
**Mechanical ventilation, No. (%)**	27(96.4%)	10(90.9%)	0.49
**ICU admission, No. (%)**	26(96.3%)	11(100%)	1
**Outcome, No. (%)**			1
Recovery	23(82.1%)	9(81.8%)	
Death	5(17.9%)	2(18.2%)	

Comparison of characteristics, MG treatment, TTC features, supportive therapy, and outcomes between patients with apical and non-apical TTC

The median age and sex distribution were broadly similar between the apical and non-apical groups. Pre-TTC MG treatments and left ventricular ejection fraction categories were also generally comparable between groups.

Median troponin levels appeared numerically higher in the apical group (2.47 ng/mL, IQR: 2.927) than in the non-apical group (0.814 ng/mL, IQR: 1.55); however, given the small sample size and limited statistical power, this observation should be interpreted cautiously and considered hypothesis-generating rather than indicative of a true subgroup difference.

Overall, trigger patterns, treatments for myasthenic exacerbation, need for circulatory support, mechanical ventilation, ICU admission, and outcomes were broadly similar between groups.

## Discussion

This systematic review provides an updated, comprehensive analysis of TTC in patients with MG. Based on our search, which retrieved 2,241 records from PubMed, Web of Science, and Google Scholar, we identified a high mortality rate of 17.5% in the collected cases. This significantly exceeds the mortality reported for isolated MG or TTC [2, 5]. While a similar review was published in 2019, our study adds critical value by incorporating newer cases and providing the first comparative analysis between apical and non-apical subtypes [7, 51]. Notably, we found that myasthenic crisis, rather than emotional stress, is the primary trigger for TTC in this population, regardless of sex.

The “synergistic lethality” observed in this study likely stems from a catecholamine-mediated “vicious circle” between neuromuscular failure and cardiac dysfunction, consistent with prior reports describing hypothalamic–pituitary–adrenal axis activation and catecholamine surges as central mechanisms in stress-induced cardiomyopathy [52]. MG crises induce severe physiological stress and hypoxia, which are potent triggers for the supraphysiological catecholamine release that precipitates TTC. Conversely, the acute left ventricular dysfunction in TTC can further impair systemic perfusion and exacerbate respiratory muscle weakness, complicating the management of MG and leading to the observed high mortality. This shared pathway of autonomic dysregulation suggests that TTC in MG is not merely a comorbid event, but a direct consequence of the physiological strain imposed by neuromuscular failure [10, 53].

### Clinical implications

These findings have important clinical implications. Because chest pain was frequently absent, clinicians should not rely on classic cardiac symptoms when evaluating patients with MG exacerbation. Respiratory distress should not be assumed to be purely neuromuscular, and routine ECG and cardiac biomarker screening should be considered during MG crisis to facilitate early detection of TTC. Early recognition of cardiac involvement is critical, given the high observed mortality.

### Subtype analysis and statistical considerations

In the comparison between TTC subtypes, no statistically significant differences were observed between apical and non-apical types. However, numerically lower troponin levels and higher corticosteroid use were observed in the non-apical group. Given the small sample size and case-report-based design, these observations should be interpreted with caution and considered hypothesis-generating rather than indicative of true subtype-specific differences. Further investigation in larger, prospective cohorts is warranted.

### Limitations

This study has several limitations. First, because this review is based on case reports and case series, there is an inherent risk of selection bias favoring the publication of severe or unique cases. Furthermore, we identified potential reporting bias during the quality assessment using CARE guidelines, characterized by missing clinical information or selective reporting in some instances. It is also possible that mild cases of TTC, especially those presenting primarily with respiratory symptoms in the context of MG crisis, were underdiagnosed or underreported, particularly when chest pain was absent or echocardiography was not performed. Thus, our dataset may overrepresent severe, easily diagnosable cases. Second, there was variability in the quality and completeness of case descriptions, and some data points were missing. Moreover, the reliance on case reports with variable reporting completeness may have introduced classification bias, particularly in differentiating TTC subtypes. The lack of standardized diagnostic and therapeutic protocols also limited the ability to compare interventions quantitatively. Third, although non-English reports were included, translations were performed using a combination of machine and expert translation. Therefore, the potential for misinterpretation of clinical details cannot be excluded entirely. Finally, and most importantly, because this review is based exclusively on case reports, inferential statistical testing is of limited validity and may be misleading. The study is clearly underpowered to detect meaningful differences between subgroups, as reflected by many p-values approaching 1.0. Therefore, all statistical analyses must be regarded as descriptive and hypothesis-generating rather than confirmatory. These results should not be interpreted as evidence of true equivalence or the absence of clinical differences between TTC subtypes. Publication bias toward severe or dramatic presentations cannot be excluded and may have contributed to the observed mortality rate.

### Future directions

Future multicenter prospective studies explicitly focused on MG–TTC co-occurrence are needed to clarify the underlying pathophysiological mechanisms and establish standardized treatment strategies. Specific investigation into the clinical significance of non-apical TTC variants, their association with echocardiographic and biomarker profiles, and the impact of combined immunotherapy and circulatory support on patient outcomes is warranted.

## Conclusions

In this systematic review, we comprehensively collected and analyzed published case reports of TTC associated with MG, focusing on clinical characteristics, treatments, and outcomes. Additionally, we compared apical and non-apical types of TTC to examine differences between subtypes.

Among the included cases, TTC in MG patients predominantly occurred in elderly females (67.5%), with the apical type accounting for approximately 71.8%. Although it is generally reported that emotional stress is more common as a trigger for TTC in females and physical stress in males, the most common trigger for TTC in our review—regardless of sex—was a myasthenic crisis (37.5%). These findings should be interpreted in the context of the exploratory, case-report-based nature of the data.

## Supplementary Information

Below is the link to the electronic supplementary material.

Supplementary Material 1

## Data Availability

No datasets were generated or analysed during the current study.

## Abbreviations

MG: Myasthenia Gravis
TTC: Takotsubo Cardiomyopathy
AChR: Acetylcholine Receptor
MuSK: Muscle-Specific Receptor Tyrosine Kinase
EF: Ejection Fraction
ECG: Electrocardiogram
IVIG: Intravenous Immunoglobulin
IABP: Intra-Aortic Balloon Pumping
COPD: Chronic Obstructive Pulmonary Disease
